# Supplementary figures and images for: Subcellular proteomics of dopamine neurons in the mouse brain
Source: eLife. 2022 Jan 31;11:e70921. doi: 10.7554/eLife.70921 (PMC8860448; doi:10.7554/eLife.70921)

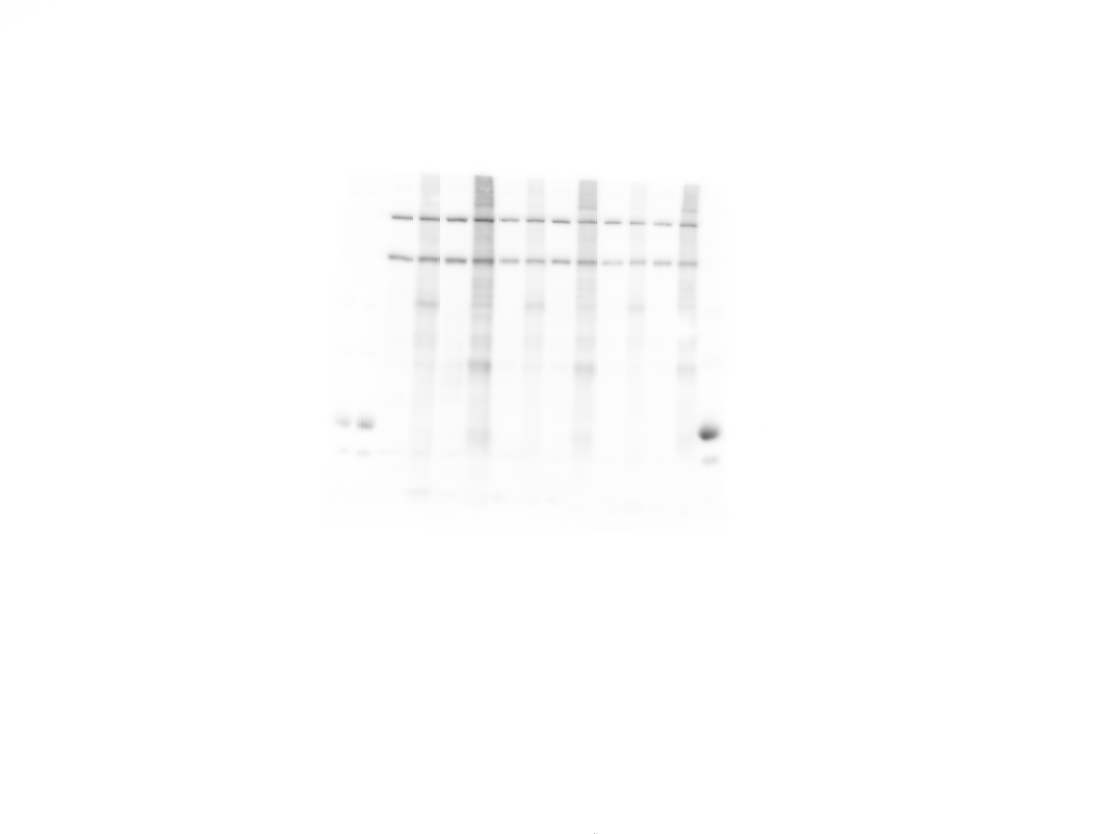

Supplement: Figure 1—source data 1. [file elife-70921-fig1-data1.zip › Figure1d_StrepHRP_Raw.tif]

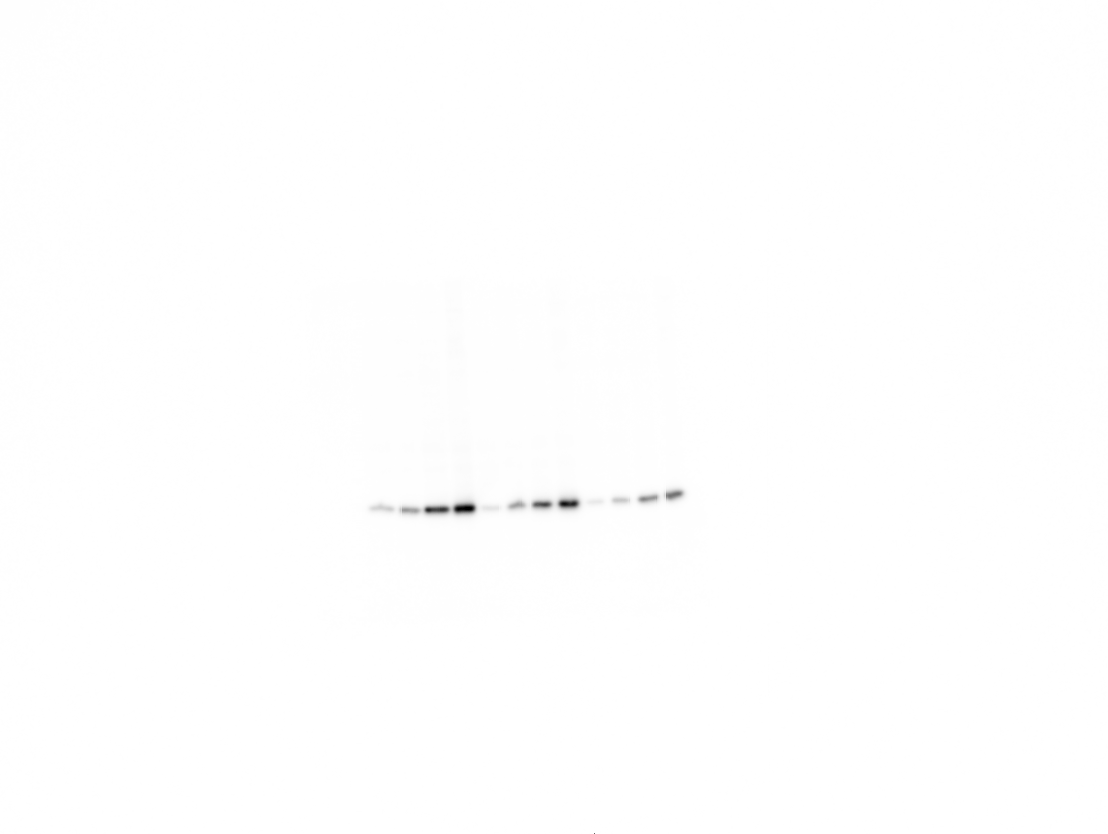

Supplement: Figure 1—source data 1. [file elife-70921-fig1-data1.zip › Figure1d_V5_Raw.tif]

## Figure 1d Western Blots

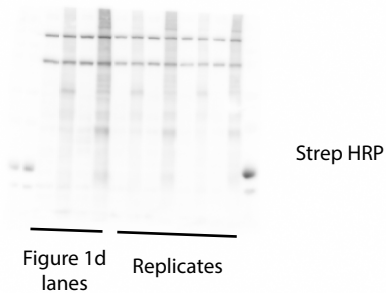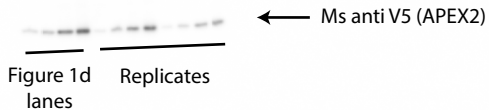

Supplement: Figure 1—source data 1. [file elife-70921-fig1-data1.zip › Figure1d_LabeledBlots.pdf]

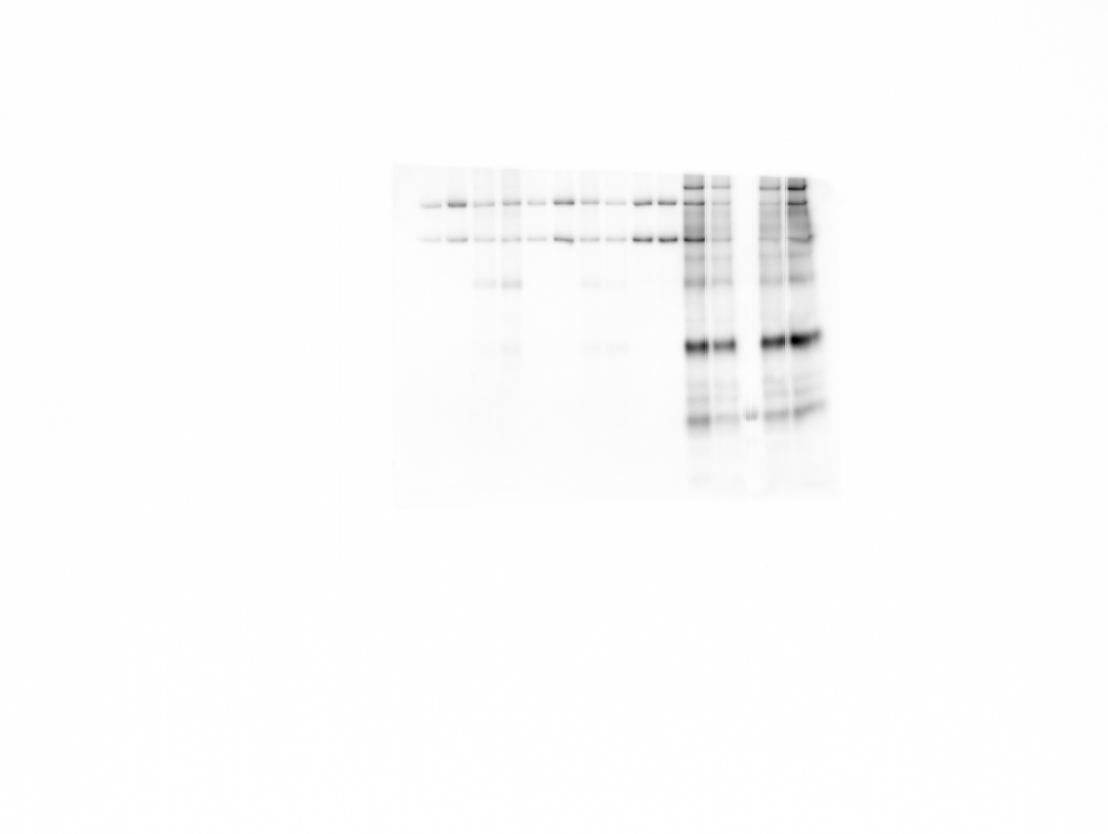

Supplement: Figure 2—source data 6. [file elife-70921-fig2-data6.zip › Figure2b_StrepHRP_Raw.tif]

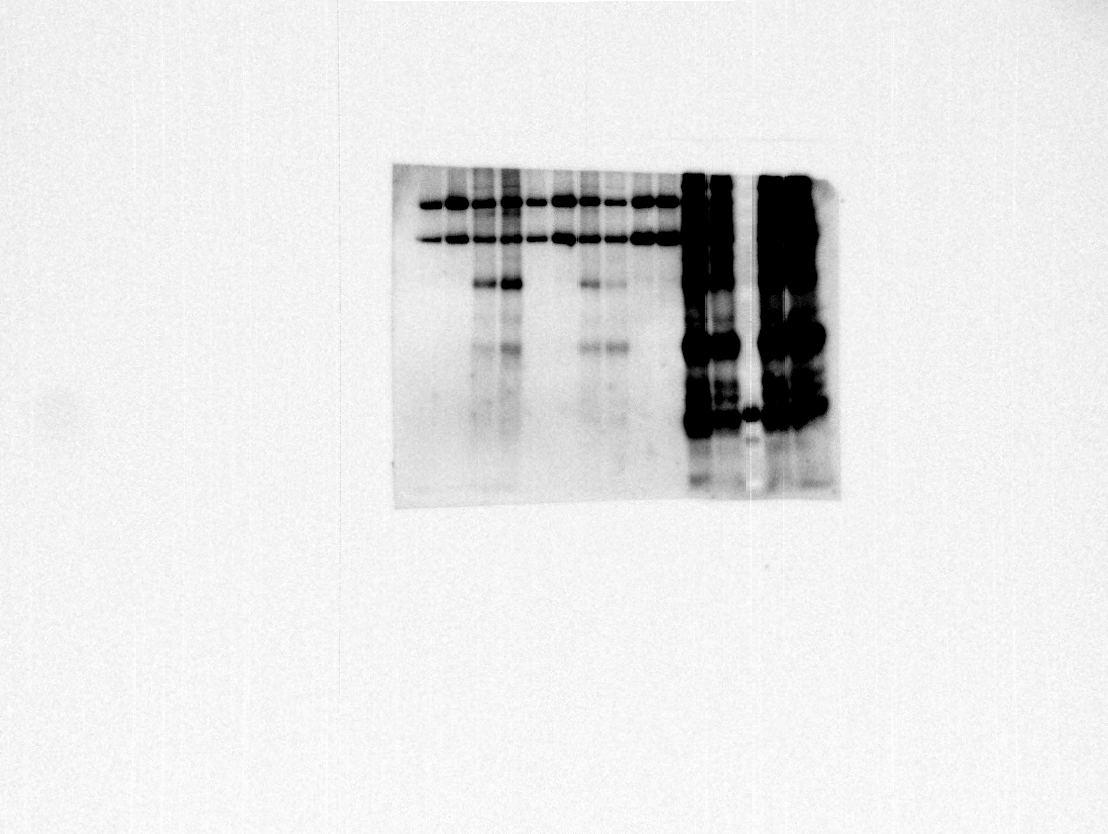

Supplement: Figure 2—source data 6. [file elife-70921-fig2-data6.zip › Figure2b_StrepHRP_HiContrast.tif]

## Figure 2b Western Blots

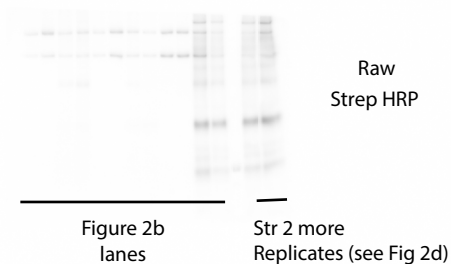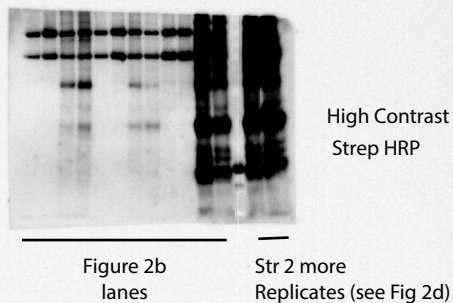

Supplement: Figure 2—source data 6. [file elife-70921-fig2-data6.zip › Figure2b_LabeledBlots.pdf]

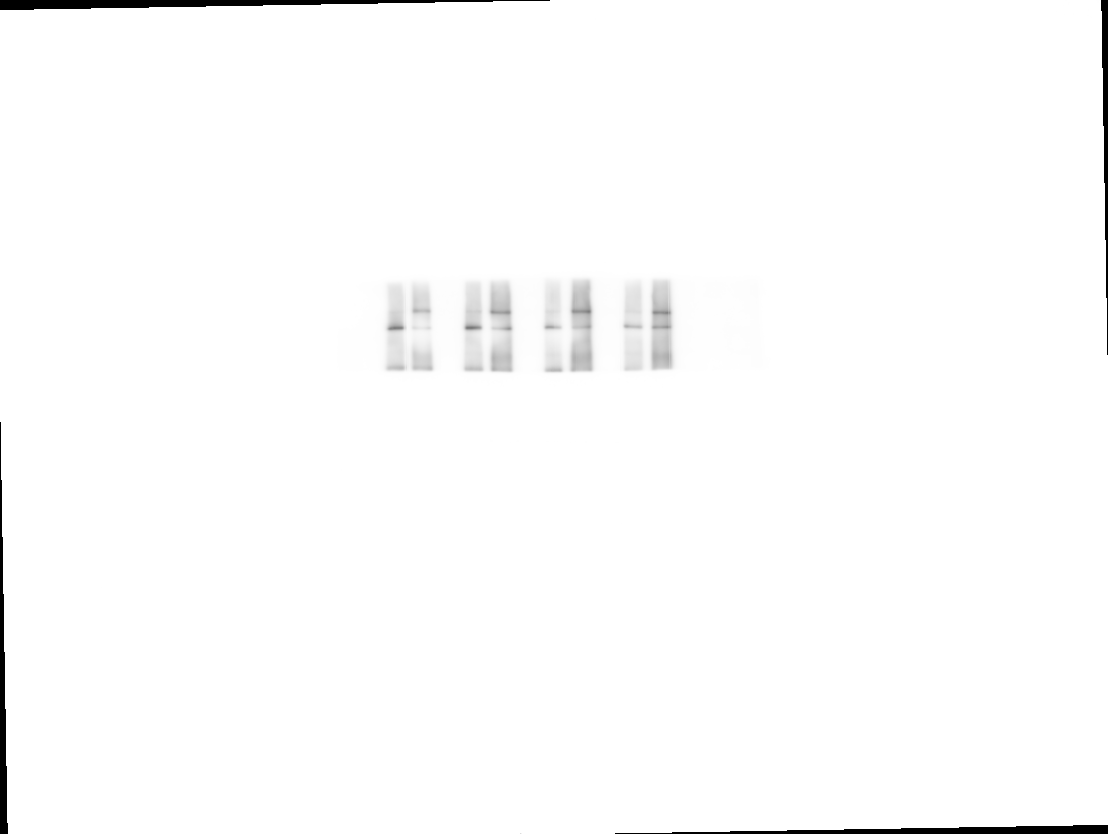

Supplement: Figure 4—source data 1. [file elife-70921-fig4-data1.zip › Figure4a_StrepHRP_Raw.tif]

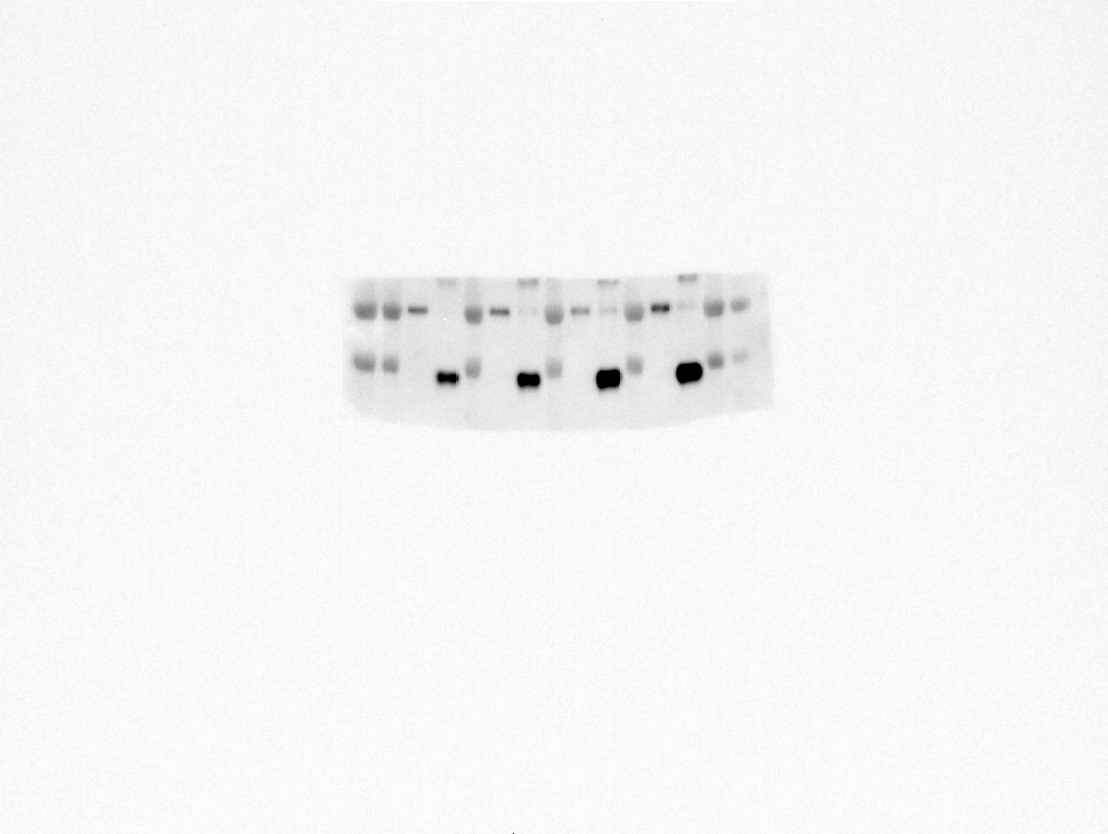

Supplement: Figure 4—source data 1. [file elife-70921-fig4-data1.zip › Figure4a_SYP-BIIItub_Raw.tif]

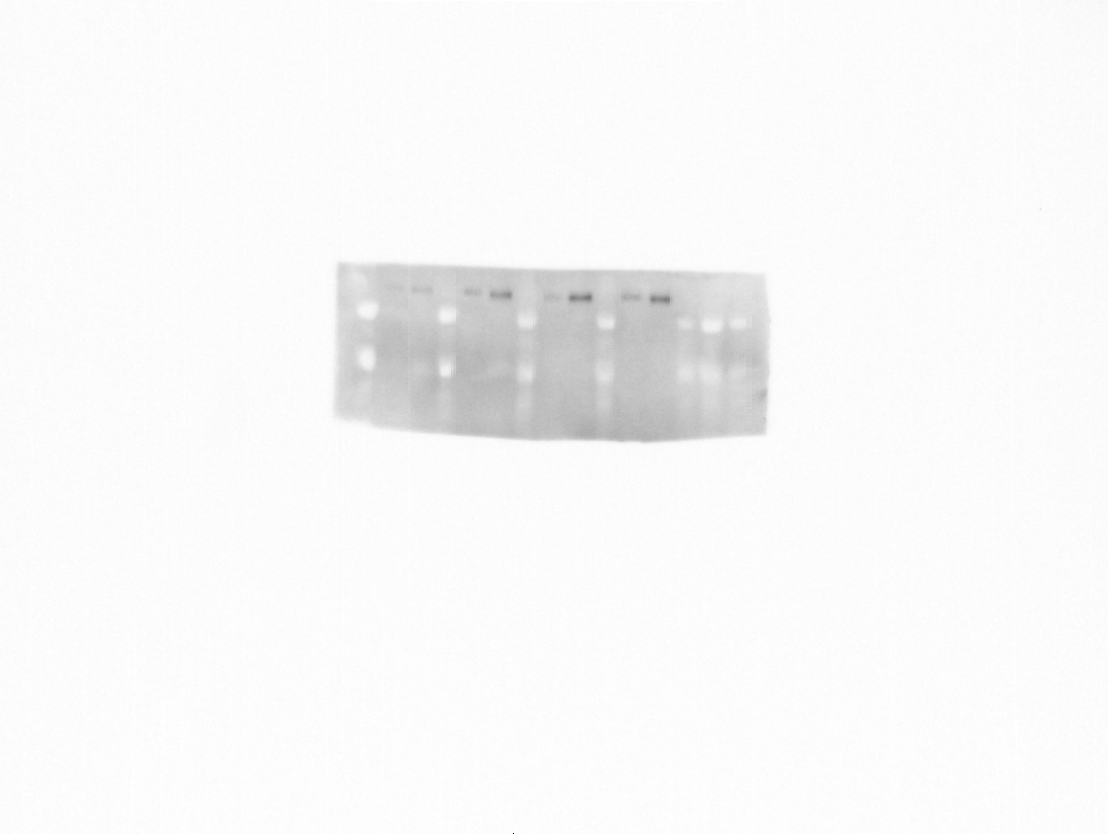

Supplement: Figure 4—source data 1. [file elife-70921-fig4-data1.zip › Figure4a_TH_Raw.tif]

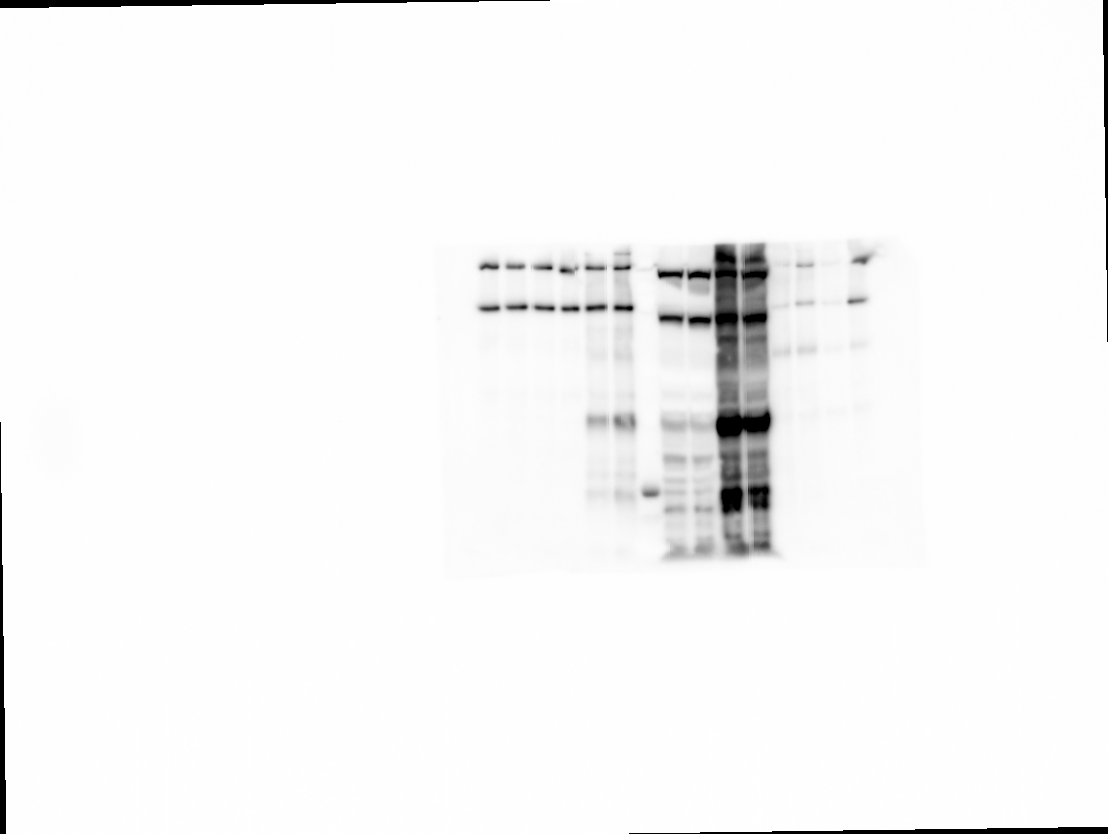

Supplement: Figure 5—source data 4. [file elife-70921-fig5-data4.zip › Figure5b_StrepHRP_HiContrast.tif]

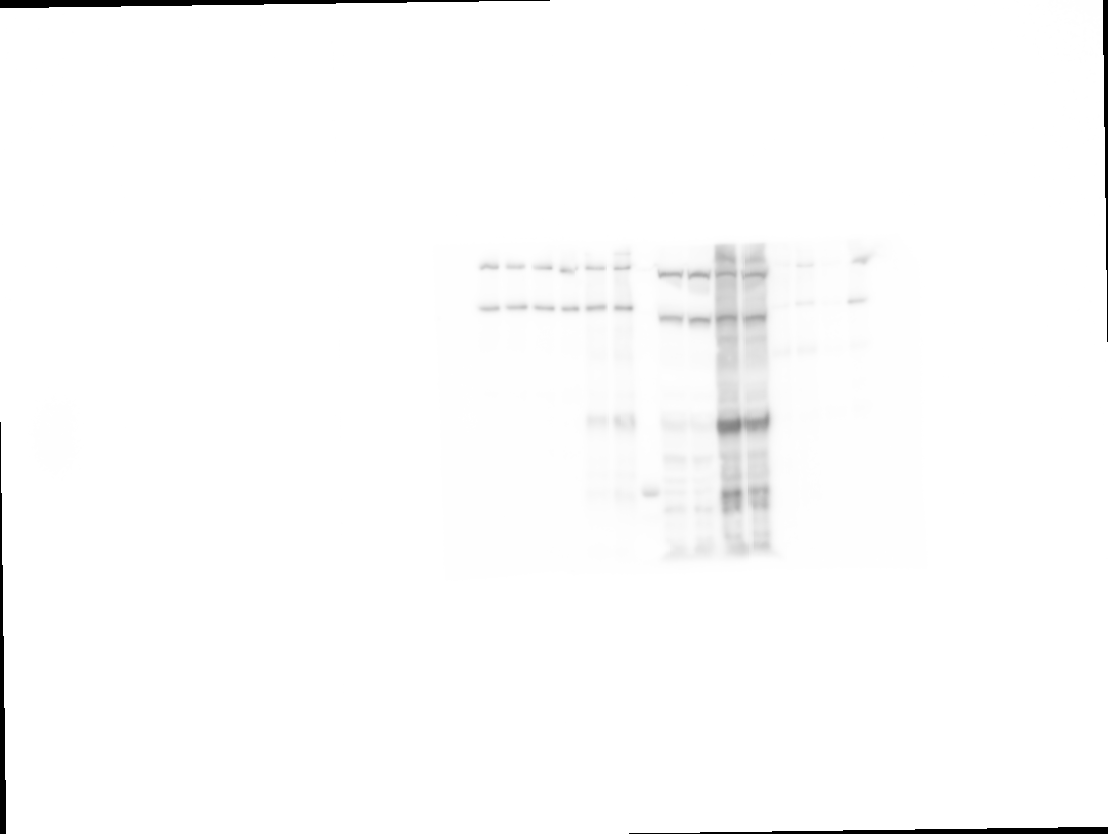

Supplement: Figure 5—source data 4. [file elife-70921-fig5-data4.zip › Figure5b_StrepHRP_Raw.tif]
