# Supplementary material for: Subcellular proteomics of dopamine neurons in the mouse brain
Source: eLife. 2022 Jan 31;11:e70921. doi: 10.7554/eLife.70921 (PMC8860448; doi:10.7554/eLife.70921)
Supplement: Figure 4—source data 1. [file elife-70921-fig4-data1.zip › Figure4a_LabeledBlots.pdf]

Figure 4a Western Blots

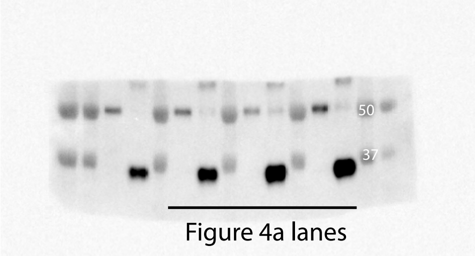

Probes
anti-BIII-tubulin, ~50 kDa
anti-synaptophysin, ~ 37 kDa

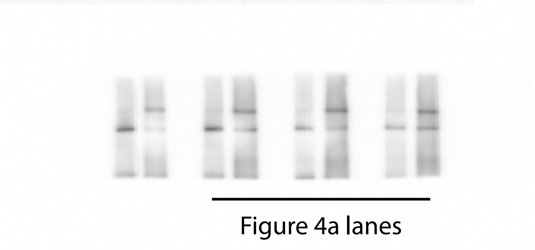

Strep HRP

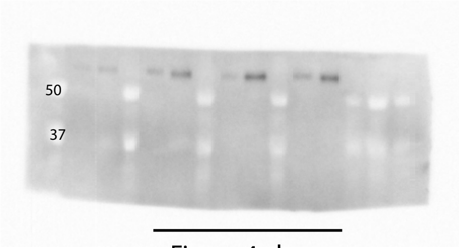

anti-Tyrosine Hydroxylase
~60 kDa
