# Supplementary material for: Subcellular proteomics of dopamine neurons in the mouse brain
Source: eLife. 2022 Jan 31;11:e70921. doi: 10.7554/eLife.70921 (PMC8860448; doi:10.7554/eLife.70921)
Supplement: Figure 5—source data 4. [file elife-70921-fig5-data4.zip › Figure5b_LabeledBlots.pdf]

Figure 5b Western Blots

Raw

Strep HRP

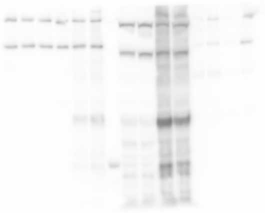

2 more Ctrls (see Fig 5d) | Figure 5b lanes | Unrelated samples

High Contrast

Strep HRP

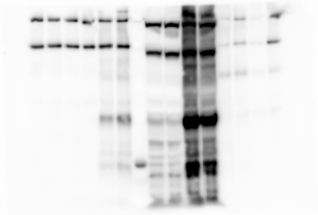

2 more Ctrls (see Fig 5d) | Figure 5b lanes | Unrelated samples
